# Algorithmic Gamification and Gig Workers’ Proactive Service Behaviour: An Attribution Theory Perspective

**DOI:** 10.3390/bs16091688

**Published:** 2026-09-19

**Authors:** Yepeng Wu, Xin Yan, Yuanyuan Jiao

**Affiliations:** 1 School of Economics and Management, Hubei University of Technology, Wuhan 430068, China; 20221040@hbut.edu.cn; 2 Hubei Digital Industrial Economy Development Center, Hubei University of Technology, Wuhan 430068, China; 3 School of Business Administration, Anhui University of Finance and Economics, Bengbu 233030, China; 4 Business School, Nankai University, Tianjin 300071, China

**Keywords:** algorithmic gamification, gig workers, proactive service behaviour, attribution theory, algorithmic procedural fairness

## Abstract

Algorithmic gamification has become an important tool for gig platforms to shape labour input; however, its relationship with the proactive service behaviour of gig workers remains unclear. Drawing on attribution theory, this study developed a dual-path model linking algorithmic gamification to gig workers’ proactive service behaviour through commitment attribution and control attribution, with algorithmic procedural fairness as a boundary condition. The results indicate that algorithmic gamification is positively associated with proactive service behaviour through commitment attribution and negatively associated with it through control attribution. Further analyses revealed that the indirect effect through commitment attribution was larger in absolute magnitude than that through control attribution, yielding a statistically significant positive total indirect effect. Moreover, algorithmic procedural fairness strengthens the positive association between algorithmic gamification and commitment attribution and attenuates its positive association with control attribution. At high levels of algorithmic procedural fairness, the conditional indirect effect of control attribution was not statistically significant. By examining workers’ attributions of platform intent, this study identifies two mechanisms linking algorithmic gamification to extra-role service behaviour and offers managerial implications for the design of algorithmic gamification on gig platforms.

## 1. Introduction

The continued success of gig platforms depends not only on gig workers completing in-role tasks, such as accepting orders and making deliveries, but also on their proactive extra-role service behaviours that exceed established requirements, such as anticipating customer needs and providing additional assistance (Z. Zhang et al., 2025). However, platform services are primarily characterised by short-term transactions and the absence of onsite interpersonal supervision (D. Wu & Huang, 2024). Moreover, algorithms have difficulty identifying and explicitly rewarding proactive services (Y. Wu et al., 2025), making it challenging for gig workers to sustain such behaviour. To this end, platforms embed game elements, including leaderboards, levels, challenge tasks, and badges, into algorithmic dispatching, performance feedback, and incentive systems, a practice known as algorithmic gamification (Bitrián et al., 2024; Yang et al., 2024). Unlike gamification in traditional organisations, which is often a discrete and voluntary intervention, algorithmic gamification is embedded in platforms’ dispatching and performance evaluation systems. Therefore, opting out or failing to meet gamified targets may reduce workers’ order opportunities and income (Cameron, 2024; Vasudevan & Chan, 2022). This combination of incentive and governance functions makes the consequences of algorithmic gamification uncertain. Although it can offer experiences of achievement and reward, it may also intensify emotional labour and anxiety through social comparison (Butler & Spoelstra, 2024) and contribute to burnout and resistance among gig workers (Santabarbara et al., 2026; Vasudevan & Chan, 2022). Therefore, understanding how algorithmic gamification affects gig workers’ proactive service behaviour is important for the sustainable development of gig platforms.

Existing research has examined gamification in contexts such as digital sales, environmental participation, and hospitality services, and largely acknowledges its positive effects on work enjoyment (Gerdenitsch et al., 2020), satisfaction (Oppong-Tawiah et al., 2020), engagement (Sesliokuyucu et al., 2026), and efficiency (Yang et al., 2024). However, evidence from gig platforms suggests that gamification may also operate as a subtle form of algorithmic control. Cameron (2024) indicated that platform-designed “efficiency games” direct workers’ attention toward income and quantified metrics, potentially crowding out difficult-to-record extra services, such as customer communication. Santabarbara et al. (2026) further found that gamification can activate controlled motivation, driving workers into self-exploitation and compulsive labour. These findings establish an important but unresolved tension in the literature. First, existing studies of algorithmic gamification have focused primarily on quantifiable in-role performance, including work involvement (Wei et al., 2025), continued participation (W. Zhang et al., 2026), and task performance (Pereira et al., 2024), whereas proactive service behaviour is anticipatory and discretionary (Rank et al., 2007), and the mechanisms established for in-role performance cannot be assumed to apply directly. Second, most studies draw on self-determination theory (Hur et al., 2022) or the job demands–resources model (Sesliokuyucu et al., 2026) to explain the effects of gamification. These perspectives remain at the level of gig workers’ perceptions of gamification’s functional affordances and thus struggle to explain why gig workers respond to identical gamification designs in diametrically opposite ways. For instance, a leaderboard may be viewed either as feedback that helps gig workers monitor their performance and demonstrate competence or as a control device that manufactures competition and intensifies surveillance. The behavioural consequences of algorithmic gamification, therefore, depend not only on its technical features but also on how gig workers infer the platform’s intent in deploying it (Bellesia et al., 2023; Zhou et al., 2025).

Attribution theory provides a framework for explaining how people infer intent. It proposes that people use observable cues to infer the intentions underlying management practices and respond to those intentions through their attitudes and behaviours (Harvey et al., 2014). This logic is particularly relevant to proactive service behaviours. Because platforms have difficulty identifying and rewarding such behaviour, gig workers’ willingness to invest additional effort depends partly on how they construe their reciprocal relationship with the platform (Liang et al., 2024). Algorithmic gamification provides task support, recognises achievement, and communicates benefits, while also tracking performance, ranking workers, and constraining behaviour. These supportive and controlling features coexist within the same algorithmic design and may simultaneously activate two distinct types of platform intent attribution with opposing effects on proactive service behaviour. Gig workers form commitment attribution when they believe that the platform uses gamification to support their development, improve their work experience, and enhance joint service performance; they form control attribution when they believe that the platform seeks to reduce management costs, intensify surveillance, or extract additional labour input (Zhou et al., 2025). Commitment attribution can foster reciprocal expectations and broaden perceived service responsibilities, whereas control attribution can encourage strategic compliance and reduce discretionary services that offer no explicit returns.

Furthermore, algorithmic procedural fairness is an important contextual cue. Algorithmic procedural fairness refers to gig workers’ perceptions of the fairness of the procedures through which platform algorithms allocate tasks, evaluate performance, and administer rewards and sanctions (Jabagi et al., 2025). High algorithmic procedural fairness signals that the platform is willing to constrain its power and consider workers’ interests, making the supportive meaning of gamification more credible and commitment attribution more likely (Morse et al., 2022; Rahman, 2021). In contrast, when algorithmic procedural fairness is low, workers are more likely to interpret algorithmic gamification as an instrument of covert platform control and thus form control attribution. Existing research on the boundary conditions of algorithmic gamification has mainly focused on tasks (Lee, 2018) and individual employee characteristics (Hammedi et al., 2021). However, little attention has been paid to whether the procedural properties of the algorithm itself provide contextual cues that calibrate workers’ attribution of the platform’s intent.

Drawing on attribution theory, this study develops and tests a dual-path model linking algorithmic gamification to gig workers’ proactive service behaviour through commitment attribution and control attribution, and examines the moderating role of algorithmic procedural fairness.

This study makes three main contributions to the literature. First, from the perspective of algorithmic gamification, it identifies an important antecedent of gig workers’ proactive service behaviour and reveals its “double-edged sword” effect. This extends research on proactive service behaviour from traditional interpersonal management contexts, such as hospitality and catering (Jiang et al., 2023) and banking (Hamzah et al., 2020), to the context of algorithmic governance (Y. Wu et al., 2025), while providing new evidence for the commitment–control paradox in gig work at the level of extra-role behaviour (Cameron, 2024; Santabarbara et al., 2026). Second, it identifies and verifies the dual mediating mechanisms of commitment attribution and control attribution, confirming that these are parallel attributional outcomes coexisting within the same gig worker (Hu et al., 2025; Nishii et al., 2008). The indirect effect of the commitment attribution path was numerically greater than that of the control attribution path, extending gamification research from the perspective of platform–intent construction. Third, whereas existing research has primarily examined the direct effects of procedural fairness on algorithmic acceptance, trust, and work attitudes (Pinho & Colston, 2025; Starke et al., 2022), this study finds that algorithmic procedural fairness strengthens the commitment attribution path and weakens the control attribution path. At high levels of algorithmic procedural fairness, the indirect effect through control attribution was not statistically significant, offering insights into how platforms can optimise algorithmic gamification design (Morse et al., 2022).

## 2. Literature Review

### 2.1. Algorithmic Gamification on Gig Platforms

Gig workers undertake tasks through online labour platforms under non-standard, temporary, or flexible working arrangements (D. Wu & Huang, 2024). Gig work is typically characterised by on-demand order acceptance, piece-rate pay, and the absence of onsite interpersonal supervision, and it is embedded in a tripartite “platform–worker–customer” relationship (Santabarbara et al., 2026). Within this structure, algorithmic systems perform many governance functions traditionally undertaken by human managers. Algorithmic management refers to the overall system in which platforms deploy algorithms to perform managerial functions such as task assignment, performance evaluation, and the administration of rewards and penalties (Duggan et al., 2023; Kellogg et al., 2020). Algorithmic gamification refers to the use of algorithms to embed game elements in task execution, performance feedback, and reward and sanction processes to steer labour and service behaviour (Deterding, 2019; Yang et al., 2024). Algorithmic gamification is a specific design practice within algorithmic management. Compared with gamification used as an auxiliary incentive in traditional organisations, algorithmic gamification is more difficult to avoid and more consequential (Kadolkar et al., 2025). Gig workers can rarely opt out of the rules of the game, and their gamified performance can directly affect order opportunities and income (Kadolkar et al., 2025). Thus, algorithmic gamification combines incentives with governance functions. Prior research has emphasised its potential benefits, including enhanced work enjoyment (Gerdenitsch et al., 2020), increased job satisfaction and engagement (Silic et al., 2020), promotion of workplace pro-environmental behaviour (Oppong-Tawiah et al., 2020), and improved work efficiency (Jacob et al., 2022). However, evidence from gig platforms presents a complex picture. Yang et al. (2024) found that gamification promotes gig workers’ customer-oriented organisational citizenship behaviour, suggesting that game elements can motivate extra-role service investment. In contrast, Cameron (2024) found that gamification may draw workers into efficiency competitions centred on income and quantified metrics, prompting them to reduce customer communication and other extra services that algorithms cannot readily record or reward. Santabarbara et al. (2026) showed that gig workers’ longer hours online and greater labour input do not necessarily reflect autonomous motivation and may instead stem from introjected motivation and a fear of missing out. This disparity suggests that the technical features of algorithmic gamification do not uniformly determine workers’ responses. Bellesia et al. (2023) identified workers’ interpretations of algorithmic arrangements as a crucial link between technological design and behavioural responses. Therefore, the consequences of algorithmic gamification may depend not only on the game elements that a platform deploys but also on how gig workers interpret the purposes behind their deployment. Overall, existing research still has the following gaps. First, existing research has examined the mechanisms of gamification mainly from an affordance perspective informed by the self-determination theory or the job demands–resources model, with limited attention paid to the attributional process through which workers infer the intent of the platform. Second, it has also concentrated on outcomes such as work involvement (Wei et al., 2025), continued participation in gamified virtual CSR activities (W. Zhang et al., 2026), task performance (Pereira et al., 2024), and workaholic behaviour (Santabarbara et al., 2026). Such findings do not adequately explain proactive service behaviour, which is spontaneous, anticipatory, and discretionary (Rank et al., 2007), and cannot readily be mandated through algorithmic rules. Third, self-determination theory and the job demands–resources model primarily explain “what gig workers experience” through gamification from a content-based perspective, but they less frequently examine how gig workers infer “why the platform adopts these designs” from a process-based perspective. Therefore, this study examines the opposing pathways through which algorithmic gamification is associated with gig workers’ proactive service behaviour.

### 2.2. Attribution Theory

Attribution theory, proposed by Heider (1958), posits that when individuals encounter uncertain events, they seek to understand and predict their social environment by inferring the causes of the observed behaviours. This attributional logic has been subsequently applied to human resource management: employees evaluate not only what a management practice entails but also why the organisation has implemented it, and they form attitudes and behavioural intentions accordingly. Nishii et al. (2008) further distinguished commitment-focused from control-focused attributions of management: the former refers to employees’ belief that the organisation implements practices to improve service quality, support employee development, or enhance employee well-being, whereas the latter refers to the belief that practices are implemented to cut costs, tighten control, or extract more labour input. This distinction provides a fundamental framework for understanding why employees react differently to the same management practices. Following this framework, this study distinguishes between commitment attribution and control attribution as two ways in which gig workers interpret the platform’s intent in implementing algorithmic gamification (Zhou et al., 2025). Prior studies have mostly drawn on self-determination theory (Hammedi et al., 2021; Yang et al., 2024) and the job demands–resources model (Sesliokuyucu et al., 2026) to investigate the consequences of gamification. Self-determination theory asks whether gamification satisfies or frustrates the need for autonomy, competence, and relatedness, whereas the job demands–resources model asks whether gamification functions as a job resource or demand. Therefore, both characterise gig workers’ experience of gamified functional affordances, treating their reactions as responses to the gamification features themselves. Consequently, these perspectives struggle to explain why objectively similar gamification designs trigger diametrically opposed psychological and behavioural responses. Attribution theory shifts the analytical focus to the sense-making stage that precedes evaluation; that is, how gig workers infer why platforms deploy gamification. This focus is particularly relevant to gig platforms. On the one hand, algorithmic gamification is characterised by high information opacity and depersonalisation (Kellogg et al., 2020; Rahman, 2021). Gig workers have difficulty directly contacting platform managers and cannot fully understand the basis for task allocation, performance evaluation, or reward and punishment decisions; they can only infer the platform’s intentions based on game rules, algorithmic feedback, and actual consequences. On the other hand, gamification outcomes are closely linked to workers’ order opportunities, income, and account privileges (Cameron, 2024). Because gig workers have much at stake, they are strongly motivated to infer the platform’s intentions. Strong motivation to infer platform–intent, combined with limited opportunities to verify it, makes platform–intent attribution a critical link between gamification design and gig workers’ behavioural responses. It should be noted that commitment attribution and control attribution are two distinct dimensions of platform intent attribution, rather than opposite poles of a single continuum. A single algorithmic gamification system can activate both attributions because its game elements convey two sets of cues: leaderboards, levels, and challenge tasks can signal support through feedback and recognition and control through evaluation and supervision. Technical features do not point to a unique platform intent. Furthermore, the relative strength of the two types of attribution is not fixed; it also depends on the information that gig workers use to infer platform intent. Kelley (1973) further pointed out that when individuals face multiple possible causes, they augment or discount the explanatory power of each cause based on additional information. Regarding algorithmic gamification, when a platform constrains its own power in an observable and costly manner, the explanation of “supportive intent” is augmented while the explanation of “control intent” is discounted; conversely, opaque rules and arbitrary enforcement grant more explanatory power to control intent (Rahman, 2021). Algorithmic procedural fairness provides diagnostic cues. The attribution process unfolds in stages. Kelley and Michela (1980) distinguished between the attribution formation process (antecedents → attribution) and the attribution consequence processes (attribution → consequences). Bowen and Ostroff (2004) further noted that the characteristics of a management system in terms of consistency and consensus determine whether employees can form clear and stable attributions regarding “why the organisation does what it does,” and such characteristics operate during the former stage. Therefore, based on attribution theory, this study analyses the mechanism of algorithmic gamification’s influence on proactive service behaviour, as well as the moderating role of algorithmic procedural fairness.

## 3. Hypothesis Development

### 3.1. The Positive Path of Algorithmic Gamification: The Mediating Role of Commitment Attribution

In gig work settings characterised by limited interpersonal supervision, algorithmic gamification can provide positive cues for task support, contribution recognition, and beneficial feedback, thereby encouraging gig workers to commit to their jobs. First, algorithmic gamification signals task support for the users. Game elements (e.g., challenge tasks, progress indicators, and real-time feedback) translate relatively ambiguous service requirements into clear and actionable goals, thereby reducing gig workers’ uncertainty in weakly supervised environments (Cardador et al., 2017). Moreover, when workers interpret algorithmically generated information as constructive feedback rather than managerial control, they report stronger intrinsic motivation and less exhaustion (Edwards et al., 2024). Gig workers who perceive gamification as helping them understand task requirements and improve their efficiency are therefore more likely to infer that the platform provides supportive resources. Second, algorithmic gamification can signal the recognition of workers’ contributions to the organisation. Leaderboards, levels, and badges not only evaluate performance but also make workers’ achievements and progress visible (Kirchner-Krath et al., 2026). When workers perceive that the platform recognises their efforts, they are more likely to infer that it values their contributions and supports their development. Third, algorithmic gamification can signal benefits for workers. Clear links between task completion, level advancement, and rewards allow workers to perceive tangible benefits and infer that gamification supports both the platform’s performance objectives and workers’ access to work opportunities and financial rewards. Therefore, we propose:

**H1.**  *Algorithmic gamification is positively related to gig workers’ commitment attribution.*

Commitment attribution shapes gig workers’ judgements about whether to invest in proactive services. Because proactive service exceeds explicitly stated task requirements, its provision depends partly on whether workers regard additional effort as justified within a reciprocal relationship (Rank et al., 2007). When gig workers believe that the platform implements gamification to support their development, improve their work experience, and enhance service quality, they are more likely to regard the platform’s service goals as legitimate and internalise them as shared objectives (Hu et al., 2025). Commitment attribution also signals that the platform values workers’ contributions and well-being. This supportive interpretation generates reciprocal expectations, transforming proactive service from an unrewarded additional burden into a voluntary response to platform support, a means of maintaining customer relationships, and an investment in shared service goals (Eisenberger et al., 2025; Zhou et al., 2025). Therefore, even when algorithms cannot fully record proactive service behaviour, gig workers who form commitment attribution are more likely to anticipate customer needs, prevent service failures, and offer additional assistance. We therefore propose:

**H2.**  *Algorithmic gamification is positively related to gig workers’ proactive service behaviour through commitment attribution.*

### 3.2. The Negative Path of Algorithmic Gamification: The Mediating Role of Control Attribution

Algorithmic gamification may simultaneously convey controlling cues to gig workers (Duggan et al., 2023). Nishii et al. (2008) argued that employees form control-focused attributions when they believe that management practices primarily serve to cut costs, tighten constraints or extract additional labour input. Accordingly, control attribution arises when gig workers interpret algorithmic gamification as an appealing but covert means of monitoring them, regulating their behaviour and extracting more labour. First, platforms continuously record service ratings, completion times, order volumes, locations, and other information and convert these data into levels and rankings. Gamified presentation does not eliminate the underlying surveillance; some gig workers treat algorithmic scores as thresholds that restrict their work opportunities and discipline their behaviour (Mitson et al., 2025). Second, algorithmic gamification can signal rule-dependence and behavioural constraints. To attain higher levels, receive more orders, or earn additional rewards, workers must continually adjust their behaviour to meet platform-defined standards such as acceptance rates, cancellation rates, and task volumes. What appears to be a voluntary choice is, in reality, confined within the platform’s preset options; opting out of the challenge may also result in the loss of opportunities and income (Cameron, 2024). Third, continuous rewards, shifting thresholds, and ranking competitions may encourage additional, unrecognised labour. When reward conditions change repeatedly and returns remain uncertain, workers are more likely to interpret gamification as a means for the platform to increase efficiency, shift risk, and extract further labour input (Santabarbara et al., 2026). Therefore, this study proposes the following hypothesis:

**H3.**  *Algorithmic gamification is positively related to the control attribution of gig workers.*

Control attribution undermines the motivational basis of gig workers’ proactive service in several ways. First, it weakens reciprocal expectations. Proactive service requires additional time, attention, and emotional effort; however, platforms have difficulty identifying and rewarding this service. When workers believe that the platform uses gamification to extract labour value, they expect their additional effort to go unmatched by commensurate returns and become less willing to provide services beyond formal task requirements. Lu et al. (2024) suggest that high-intensity algorithmic management constitutes a hindrance job demand that undermines platform riders’ commitment to service quality. Second, control attribution weakens the legitimacy of platform service norms. Workers may outwardly comply to avoid sanctions without internalising the platform’s role expectations as personally endorsed standards (Wiener et al., 2023). Third, control attribution narrows workers’ attention and encourages strategic compliance. To avoid algorithmic penalties and stabilise their income, workers may concentrate their limited resources on quantifiable indicators such as order volume, delivery punctuality, and ratings. Therefore, they may complete prescribed tasks “by the book” while curtailing discretionary efforts that yield no explicit return (Cameron, 2022). We therefore propose:

**H4.**  *Control attribution mediates the relationship between algorithmic gamification and proactive service behaviour among gig workers; that is, algorithmic gamification negatively affects proactive service behaviour through control attribution.*

### 3.3. The Moderating Role of Algorithmic Procedural Fairness

Algorithmic procedural fairness refers to gig workers’ overall judgement of whether the platform follows the principles of consistency, bias suppression, accuracy, and correctability when designing and implementing procedures for dispatching, evaluation, ranking, rewards, and sanctions (Jabagi et al., 2025). Drawing on attribution theory, this study positions algorithmic procedural fairness as a moderator of the first-stage relationship (algorithmic gamification → attributional inference) rather than the second-stage relationship (attributional inference → proactive service behaviour). First, algorithmic procedural fairness serves as a diagnostic contextual cue primarily during the formation of platform–intent attributions. Algorithmic gamification combines empowerment and monitoring, and the intentions underlying its use are often ambiguous. Faced with this ambiguity, gig workers may use procedural fairness cues (e.g., whether rules are transparent and appeals are permitted) to infer the platform’s underlying motive. Second, once an attribution is formed, procedural cues may become less central to the behavioural responses. At this stage, proactive service behaviour may depend more on reciprocal motivation, ability, and opportunity than on further inferences about platform intent.

Furthermore, when algorithmic procedural fairness is high, transparent and verifiable performance standards, stable and consistently applied rules, and effective appeal mechanisms reduce concerns regarding arbitrary platform manipulation. Leaderboards and rewards are then more likely to be regarded as valid reflections of workers’ contributions, lending credibility to the task-support, contribution-recognition, and benefit-feedback cues embedded in algorithmic gamification, and making commitment attribution more likely (Morse et al., 2022; Zhou et al., 2025). In contrast, low algorithmic procedural fairness creates a discrepancy between the positive cues of gamification and workers’ experience of opaque rules or inconsistent implementation, weakening the inference that the platform has a supportive intention.

**H5.**  *Algorithmic procedural fairness strengthens (a) the positive relationship between algorithmic gamification and gig workers’ commitment attribution and (b) the positive indirect relationship between algorithmic gamification and gig workers’ proactive service behaviour through commitment attribution.*

Algorithmic procedural fairness also moderates the relationship between algorithmic gamification and gig workers’ control attribution. Algorithmic gamification readily evokes control attribution, partly because algorithmic monitoring systems can create a form of “one-way empowerment” (Zhou et al., 2025). By giving workers opportunities to obtain information, question decisions, and lodge appeals, high algorithmic procedural fairness partially counters this one-way power structure. This demonstrates the platform’s willingness to accept scrutiny and constrain its own power, thereby signalling less controlling intent. Therefore, high algorithmic procedural fairness should reduce the likelihood that workers will interpret algorithmic gamification as covert labour control (Martin & Waldman, 2023). In contrast, when procedural fairness is low, the “black box” character of algorithms and the one-sided power structure amplify the controlling cues embedded in the gamification. Inconsistent rule enforcement, opaque performance evaluation, and the absence of appeal channels reinforce the meanings conveyed by ranking competitions and behavioural constraints, making workers more likely to infer that the platform uses gamification to exercise covert labour control and shift risk (van Zoonen et al., 2026). We therefore propose:

**H6.**  *Algorithmic procedural fairness weakens (a) the positive relationship between algorithmic gamification and gig workers’ control attribution and (b) the negative indirect relationship between algorithmic gamification and gig workers’ proactive service behaviour through control attribution.*

**Figure 1:**
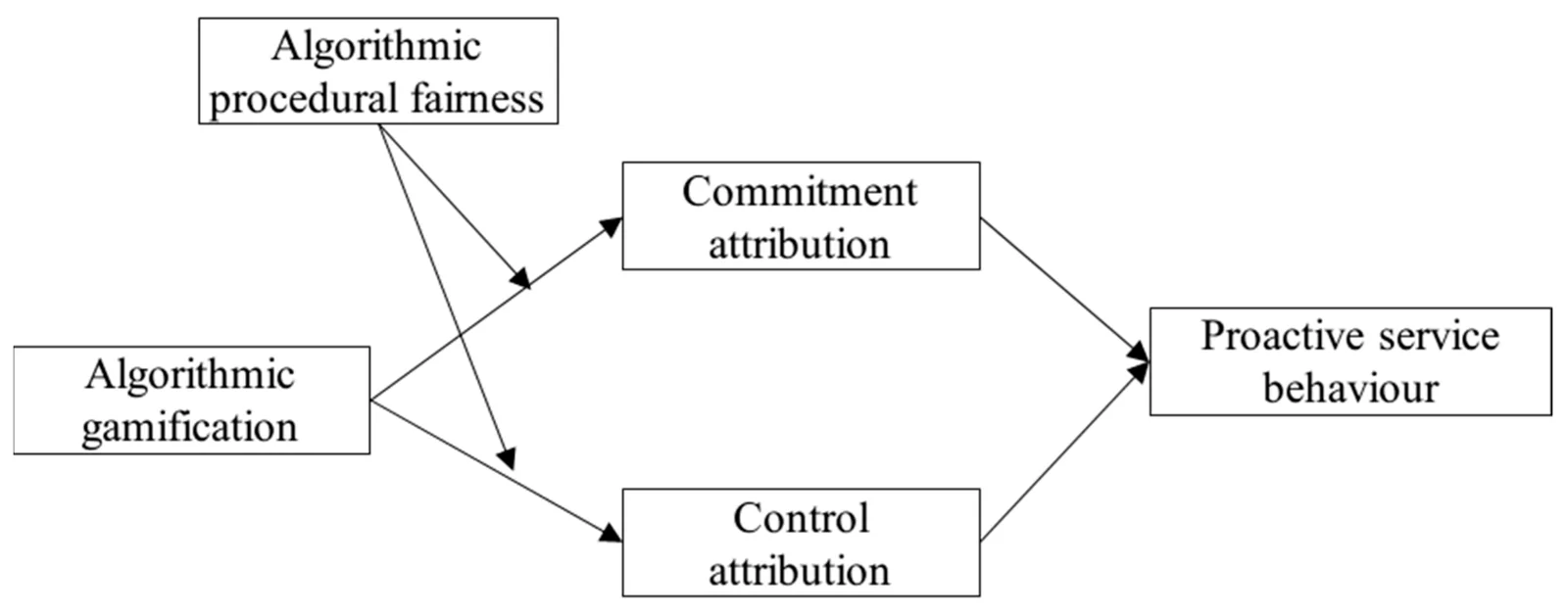
Research model.

The proposed research model is presented in Figure 1.

## 4. Research Design

### 4.1. Sample and Data

Sample and context: This study focused on food-delivery riders, ride-hailing drivers, and crowdsourced instant-delivery couriers. According to the classification of gig work by Duggan et al. (2023), these three groups all belong to “app-work.” The selection of these groups was primarily based on the following considerations: (1) The task allocation, process supervision, performance evaluation, and reward/punishment management for these types of gig workers are highly dependent on platform algorithms. (2) All three types of platforms have embedded highly isomorphic gamification mechanisms throughout the entire task management process—such as the “Rider Level + Order Challenges + Volume Leaderboards + Tiered Rewards” of food delivery platforms, the “Service Score + Driver Level + Peak Hour Rewards + Completion Rankings” of ride-hailing platforms, and the “Level Badges + Order Grabbing Challenges + King of Orders Leaderboard + Tiered Completion Bonuses” of instant-delivery platforms. These include core game elements such as leaderboards, levels, challenge tasks, progress feedback, and tiered rewards, all of which are directly linked to dispatch weighting and workers’ income. (3) All three types of work involve substantial customer interaction. Whether gig workers can proactively identify customer needs, prevent service issues, and provide extra assistance directly impacts the customer experience and platform service quality.

Sampling and recruitment: The sample for this study was collected using the Credamo platform. Before the formal survey, multiple screening criteria were established: (1) participants must currently be active in taking orders on their respective platforms and have been in the industry for no less than one month; (2) their platforms must feature gamification elements such as leaderboards, levels, challenge tasks, progress feedback, or tiered rewards (confirmed through the screening question: “Does your platform feature leaderboards, levels, challenge tasks, progress feedback, or tiered rewards?” Respondents who answered “No” or “Unsure” were excluded). Additionally, one attention check item was embedded in the questionnaire (e.g., “Please select “Strongly Agree” for this item”), and those who failed to pass were excluded.

Survey procedure: The survey used a three-wave time-lagged design with one week between consecutive waves. Each respondent received a ¥10 RMB reward for completing all three rounds of the survey. To match responses across waves without collecting personally identifiable information, each respondent was assigned a unique anonymous code in the first wave, which was retained in the next two waves. At Time 1 (T1), we measured algorithmic gamification, algorithmic procedural fairness, and demographic variables; 500 questionnaires were distributed, and 432 valid responses were obtained (response rate = 86.4%). At Time 2 (T2), we measured commitment attribution and control attribution and successfully matched 404 responses (retention rate = 93.5% relative to T1). At Time 3 (T3), we measured proactive service behaviour. After excluding responses that could not be matched across the three waves, had missing values for key variables, or displayed clearly anomalous response patterns, the final analytic sample comprised 392 complete three-wave cases, equivalent to 78.4% of the 500 questionnaires that were initially distributed. Table 1 presents the characteristics of the sample.

### 4.2. Measurement

The measurement items in this study were derived from previously established scales. The scales for algorithmic gamification and algorithmic procedural fairness were adapted to the specific platform-management context examined in this study. Two researchers in organisational behaviour independently mapped the core semantic elements of the original scale items against the corresponding management behaviours of the gig platforms. Two scholars in the field of human resource management were invited to evaluate the content validity of each item, and revisions were made based on their feedback. The English scales were translated using a translation–back–translation procedure (Brislin, 1970), and postgraduate students specialising in English checked their semantic equivalence and linguistic accuracy. All items were scored on a 5-point Likert scale.

Algorithmic gamification: We measured algorithmic gamification using a nine-item scale developed by Yang et al. (2024), which we adapted to the gig and work-on-demand contexts. Cronbach’s α was 0.950.

Commitment attribution and control attribution: We measured gig workers’ commitment attribution and control attribution regarding the platform’s implementation of algorithmic gamification using three items for each construct, adapted from Nishii et al.’s (2008) human resource attribution scale. Their Cronbach’s α values were 0.869 and 0.937, respectively, indicating good reliability.

Proactive service behaviour: We assessed proactive service behaviour using the seven-item scale developed by Rank et al. (2007). Cronbach’s α was 0.913. Self-reported measures were adopted in the context of the gig platform based on the following considerations: First, proactive service behaviour is characterised by proactivity and discretion, involving implicit components that are difficult for external observers to detect, such as anticipating customer needs and preventing service issues; therefore, the actors themselves possess the most complete information (Conway & Lance, 2010). Second, gig work lacks onsite supervisors, making supervisory evaluations unavailable. Furthermore, interactions between customers and gig workers are mostly one-off and brief; ratings from a single transaction fail to reflect a worker’s stable behavioural tendencies across multiple orders, and star ratings are often confounded by factors beyond the worker’s control, such as food quality, waiting times, and road conditions.

Algorithmic procedural fairness: This study used a seven-item procedural fairness scale adapted from Colquitt (2001). The items were contextually adapted to assess whether the platform’s algorithms adhered to the principles of consistency, unbiasedness, accuracy, and appealability throughout the processes of order dispatching, performance evaluation, rewards and punishments, and grievance feedback. Cronbach’s α was 0.911.

Control variables: Following the literature (Y. Wu et al., 2025; Zhou et al., 2025), this study included gender, age, platform type, education, tenure, daily working hours and monthly income as control variables.

The detailed measurement items and factor loadings are provided in Appendix A.

### 4.3. Results Analysis

#### 4.3.1. Measurement Model Test

We used SPSS 22.0 and AMOS 26.0 to assess the measurement reliability and validity (Table 2). The five-factor baseline model fit the data well (χ^2^ = 503.712, df = 367, χ^2^/df = 1.373, CFI = 0.983, TLI = 0.982, SRMR = 0.033, RMSEA = 0.031) and fitted the data significantly better than the alternative four-factor models (a) and (b), the three-factor model, and the one-factor model (all Δχ^2^ tests were significant at *p* < 0.001). Moreover, the square root of the AVE for each of the five focal constructs exceeded its correlation with all other focal constructs (Table 3) (Fornell & Larcker, 1981). Furthermore, the heterotrait–monotrait ratio (HTMT) values ranged from 0.052 to 0.687, all below the threshold of 0.85 (Henseler et al., 2015), providing further support for discriminant validity.

#### 4.3.2. Common Method Bias

Although this study adopted a three-wave time-lagged design, all five core variables were self-reported by gig workers, necessitating an assessment of common method bias (CMB). Following Podsakoff et al. (2003), this study introduced an unmeasured latent method factor to the five-factor baseline model, allowing all observed items to load simultaneously on their respective trait and method factors. The results showed that the fit indices after adding the common method factor were χ^2^/df = 1.275, CFI = 0.989, TLI = 0.986, SRMR = 0.025, and RMSEA = 0.027. Compared with the five-factor baseline model (CFI = 0.983, RMSEA = 0.031), the improvements in all fit indices were extremely small (ΔCFI = 0.006 < 0.01; ΔRMSEA = −0.004 < 0.01) and failed to meet the criteria for substantial improvement. Furthermore, this study employed the marker variable technique proposed by Lindell and Whitney (2001) to perform partial correlation adjustments on the correlation matrix. The results indicated that after controlling for the potential common method variance represented by the marker variable, the partial correlations between algorithmic gamification and commitment attribution (*r_A_* = 0.589, *t* = 14.39, *p* < 0.001), algorithmic gamification and control attribution (*r_A_* = 0.261, *t* = 5.35, *p* < 0.001), and other core paths remained highly significant at the 0.001 level. Taken together, these tests suggest that common method bias was not a major concern in this study.

#### 4.3.3. Descriptive Statistics and Correlation Analysis

Table 3 presents the means, standard deviations, and Pearson correlation coefficients for each variable. Algorithmic gamification was significantly and positively correlated with commitment attribution (*r* = 0.622, *p* < 0.001), control attribution (*r* = 0.320, *p* < 0.001), and proactive service behaviour (*r* = 0.474, *p* < 0.001). Commitment attribution was significantly and positively correlated with proactive service behaviour (*r* = 0.461, *p* < 0.001), whereas the zero-order correlation between control attribution and proactive service behaviour was not significant (*r* = −0.035, *p* = 0.496). The maximum variance inflation factor (VIF) in the hierarchical regression models, including the control dummy variables, was 7.431, which did not exceed the empirical threshold of 10. There was a significant positive correlation between commitment attribution and control attribution (*r* = 0.184, *p* < 0.001); however, the correlation coefficient was notably lower than the square root of the average variance extracted (AVE) for each, supporting the discriminant validity between the two attribution types.

#### 4.3.4. Hypothesis Testing

We used hierarchical OLS regression to estimate the mediation and moderated mediation models specified by PROCESS Models 4 and 7 (Hayes, 2013). Given that gender, work platform, age, education level, years of work experience, average daily working hours, and monthly income were measured in categorical or ordinal intervals with unequal widths and open ends, they were not suitable to be treated as equidistant continuous variables. Following Cohen et al. (2013), this study treats them as categorical variables and employs *K* − 1 dummy variable coding, generating a total of 19 dummy variables that were incorporated into all regression models. Before constructing the interaction terms, algorithmic gamification and algorithmic procedural fairness were mean-centred based on the full sample of 392 cases, retaining full numerical precision.

As shown in Table 4, after including the control variables, algorithmic gamification significantly and positively predicted commitment attribution (M2, *b* = 0.734, *p* < 0.001) and control attribution (M6, *b* = 0.436, *p* < 0.001), supporting Hypotheses 1 and 3. The total effect of algorithmic gamification on proactive service behaviour was significantly positive (M10, *b* = 0.500, *p* < 0.001). When both types of attributions were included in the model, commitment attribution positively predicted proactive service behaviour (M11, *b* = 0.232, *p* < 0.001), while control attribution negatively predicted proactive service behaviour (M11, *b* = −0.145, *p* < 0.001). The direct effect of algorithmic gamification remained significant (M11, *b* = 0.393, *p* < 0.001).

To test the two mediating pathways, this study followed the parallel mediation setup of PROCESS Model 4, constructing percentile confidence intervals through 5000 bootstrap resamples. The results showed that the specific indirect effect of algorithmic gamification on proactive service behaviour via commitment attribution was 0.170, with a 95% bootstrap CI of [0.083, 0.248]. The specific indirect effect of control attribution was −0.063, with a 95% bootstrap CI of [−0.103, −0.028]. As neither confidence interval included zero, Hypotheses 2 and 4 were supported. The zero-order correlation between control attribution and proactive service behaviour was not significant (see Table 3); however, after controlling for algorithmic gamification, commitment attribution, and demographic variables, a significant negative relationship was observed. This indicates a suppression effect (MacKinnon et al., 2000), highlighting the need to distinguish between the two types of attribution pathways.

**Figure 2:**
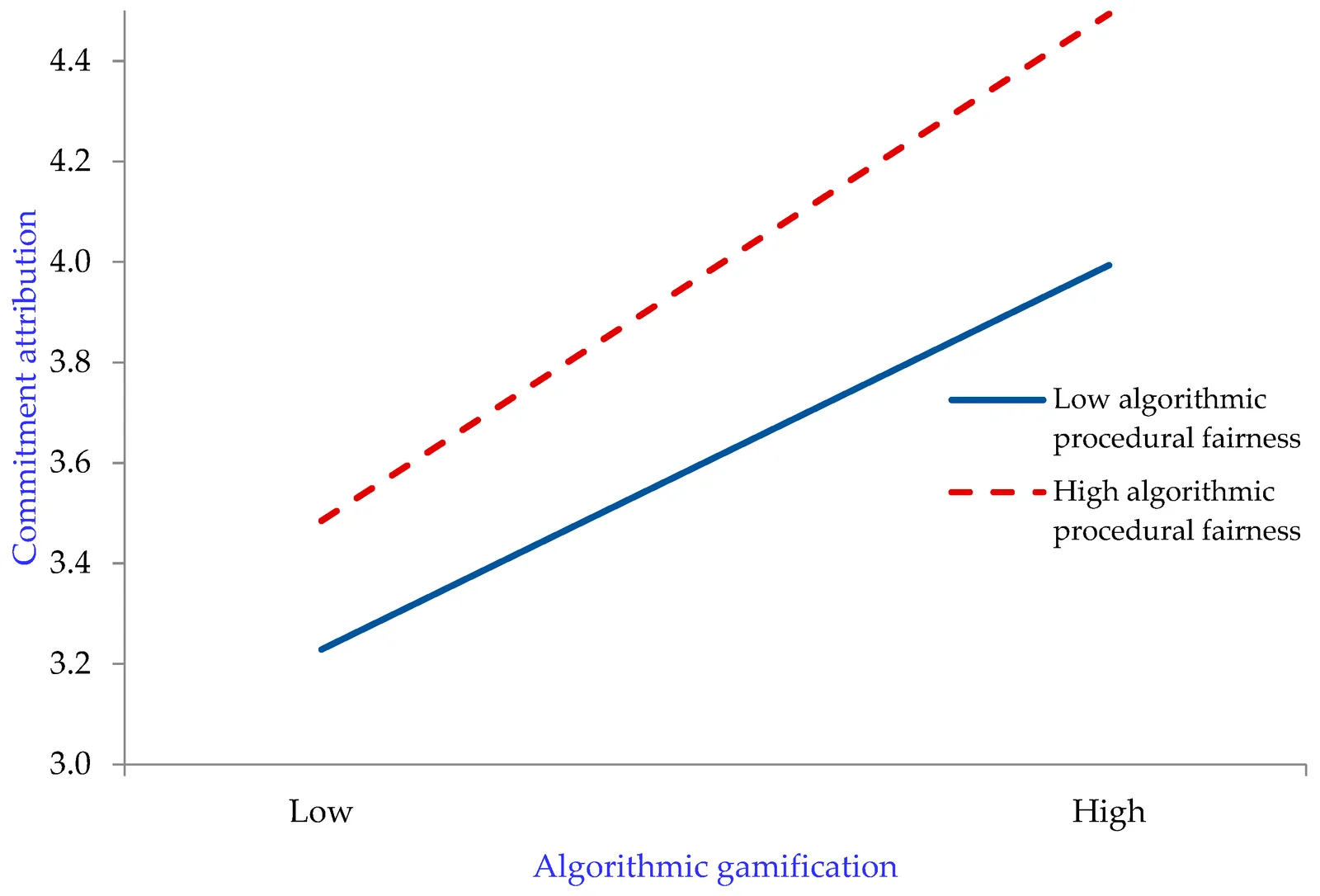
The moderating effect of algorithmic procedural fairness on the relationship between algorithmic gamification and commitment attribution.

**Figure 3:**
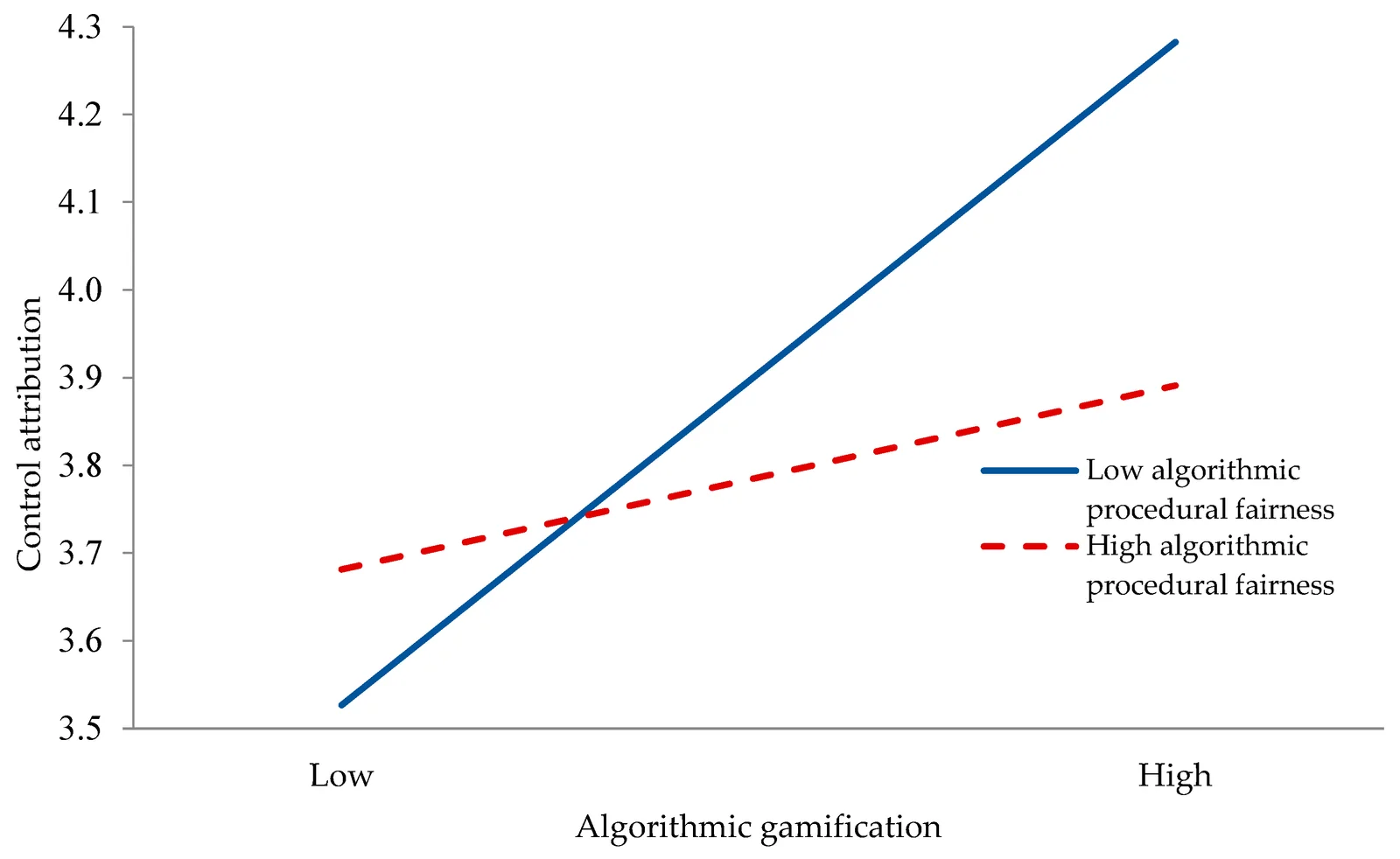
The moderating effect of algorithmic procedural fairness on the relationship between algorithmic gamification and control attribution.

The results of the moderation effect test indicated that the interaction between algorithmic gamification and algorithmic procedural fairness positively predicted commitment attribution (M4, *b* = 0.112, *p* = 0.032) and negatively predicted control attribution (M8, *b* = −0.296, *p* < 0.001), supporting Hypotheses 5(a) and 6(a). Following the procedure described by Cohen et al. (2013), this study plotted moderation effect diagrams (see Figure 2 and Figure 3) to visually present the relationship between algorithmic gamification and the two types of attribution under different levels of algorithmic procedural fairness (M ± 1 SD). Figure 2 shows that when algorithmic procedural fairness was high (M + SD), the simple slope of the effect of algorithmic gamification on commitment attribution was 0.726 (SE = 0.076, *p* < 0.001, 95% CI [0.576, 0.876]); when it was low, it was 0.574 (SE = 0.056, *p* < 0.001, 95% CI [0.464, 0.684]). Figure 3 shows that the simple slope of algorithmic gamification on control attribution was 0.554 at the low level (SE = 0.089, *p* < 0.001, 95% CI [0.379, 0.730]) and decreased to 0.154, which was not significant at the high level (SE = 0.121, *p* = 0.205, 95% CI [−0.085, 0.392]).

#### 4.3.5. Supplementary Analyses

To compare the relative strengths of the two parallel mediation paths, this study examined the total indirect effect alongside the two specific indirect effects (Table 5). In a parallel mediation model, the total indirect effect is the sum of each specific indirect effect (Hayes, 2013). Since the specific indirect effect via commitment attribution is positive (0.170) and the specific indirect effect via control attribution is negative (−0.063), the total indirect effect of 0.107 (=0.170 + [−0.063]) is not the combined strength of the two paths, but rather the net result of two opposing effects cancelling each other out; numerically, it equals the difference between their absolute values (|0.170| − |−0.063| = 0.107). This result is consistent with the decomposition of the total effect in Table 4. Given that the two paths had opposite signs, the 95% bootstrap confidence interval for the total indirect effect was [0.008, 0.193], which did not include zero. Therefore, the negative path through control attribution partially offsets but does not eliminate the positive path through commitment attribution, resulting in a net positive indirect association between algorithmic gamification and proactive service behaviour.

To test the moderating effect of algorithmic procedural fairness on the aforementioned indirect effects, this study conducted a moderated mediation test using 5000 bootstrap resamples (Model 7) (Hayes, 2013). The model simultaneously estimated two mediating paths—commitment attribution and control attribution—and included the same 19 control dummy variables in all equations. Interaction terms were constructed after centring algorithmic gamification and algorithmic procedural fairness based on the full-sample mean, and both types of attributions were included in the outcome equations. As shown in Table 6, at low (M − SD), medium (M), and high (M + SD) levels of algorithmic procedural fairness, the conditional indirect effects via commitment attribution were 0.133, 0.151, and 0.169, respectively, with 95% bootstrap CIs of [0.064, 0.198], [0.074, 0.221], and [0.082, 0.250], none of which contained zero. The index of moderated mediation for the commitment attribution path was 0.026, with a 95% bootstrap CI of [0.001, 0.063], supporting Hypothesis 5(b). The conditional indirect effects via control attribution were −0.080, −0.051, and −0.022, with 95% bootstrap CIs of [−0.129, −0.039], [−0.093, −0.017], and [−0.067, 0.017], respectively. The index of moderated mediation for this path was 0.043, with a 95% bootstrap CI of [0.019, 0.080], which did not contain zero, thus supporting Hypothesis 6(b). This indicates that as algorithmic procedural fairness increases, the negative indirect effect of control attribution diminishes.

#### 4.3.6. Multi-Group Comparative Analysis

To systematically examine the differences among the three types of work platform samples, this study conducted multi-group comparisons sequentially at the mean, measurement, and structural path levels.

This study conducted a one-way ANOVA using the work platforms as grouping variables. The results showed that the mean differences across the three types of platforms were not significant for algorithmic gamification (F(2, 389) = 1.651, *p* = 0.193, *η*^2^ = 0.008), control attribution (F(2, 389) = 1.043, *p* = 0.353, *η*^2^ = 0.005), and proactive service behaviour (F(2, 389) = 1.337, *p* = 0.264, *η*^2^ = 0.007). Food-delivery riders’ commitment attribution and perceived algorithmic procedural fairness were lower than those of ride-hailing drivers (Games-Howell *p* < 0.001, *d* = 0.65; *p* = 0.002, *d* = 0.42), while the differences compared with crowdsourced couriers were not significant. Following the criteria of Cohen et al. (2013), both were small effect sizes. This indicates that the samples from the three types of platforms are generally similar in terms of the levels of core variables, with only the food-delivery riders having relatively lower commitment attribution to platform algorithmic gamification and perceived procedural fairness.

Measurement invariance testing. A multi-group CFA was conducted using the type of work platform as the grouping variable, and the results are presented in Table 7. The results indicated that the configural invariance model fit well (χ^2^/df = 1.322, CFI = 0.958, TLI = 0.953, RMSEA = 0.050, SRMR = 0.050). Compared to the configural model, the metric invariance model showed ΔCFI = −0.0011 and ΔRMSEA = −0.0004. Compared to the metric model, the scalar invariance model, which explicitly constrains item intercepts, showed ΔCFI = −0.0015 and ΔRMSEA = −0.0002. The decreases in CFI were all less than 0.010, and RMSEA did not increase, meeting the fit change criteria suggested by Chen (2007) and supporting full scalar invariance across the three types of platforms. This indicates that the scale scores were comparable across platforms.

Cross-platform comparisons of regression paths. This study further utilised the type of work platform as a grouping variable to estimate a structural model containing the dual paths of “algorithmic gamification → commitment attribution/control attribution → proactive service behaviour” and the interaction term of “algorithmic gamification × algorithmic procedural fairness.” The results of this analysis are presented in Table 8. The results indicate that the joint tests for the commitment attribution equation (F(6,363) = 1.369, *p* = 0.226), control attribution equation (F(6,363) = 1.303, *p* = 0.255), and proactive service behaviour equation (F(6,363) = 1.424, *p* = 0.204) were all non-significant. Furthermore, the equality tests for individual paths were also non-significant (*p* ranging between 0.068 and 0.889), and the incremental explanatory power provided by the product terms was only 0.017–0.025. Using conventional OLS standard errors, the joint tests for the commitment attribution and proactive service behaviour equations were statistically significant (*p* = 0.046; *p* = 0.030, respectively), stemming from the differences in the magnitude of the coefficients for the two paths: algorithmic gamification → commitment attribution and commitment attribution → proactive service behaviour. As shown in Table 8, the directions of the conditional slopes for these two paths across the three types of platforms were entirely consistent (ranging from 0.438 to 0.857 and 0.121 to 0.423, respectively); thus, the inter-group differences are reflected in intensity rather than direction.

To further test the comparability at the model level, this study conducted stratified bootstrapping (5000 iterations) based on Model 4’s parallel mediation setup. The indirect effects via commitment attribution were positive across all three platform types (food-delivery riders: 0.178; ride-hailing drivers: 0.240; crowdsourced instant-delivery couriers: 0.113), while the indirect effects via control attribution were negative (−0.025, −0.085, and −0.060, respectively). For both pathways, the 95% confidence intervals for the pairwise differences between the three groups contained zero, indicating no significant inter-group differences. Overall, the results supported measurement comparability and consistent path directions across the three platform groups, with no statistically significant differences in indirect effects.

#### 4.3.7. Robustness Check

To assess whether algorithmic procedural fairness moderated the second-stage paths, we estimated a model with moderation at both stages using the regression equations of PROCESS Model 58 and the 19 control dummy variables as in the main analysis. Ordinary least squares (OLS) testing showed that the interaction between commitment attribution and algorithmic procedural fairness in the second stage was not significant (*b* = −0.124, *p* = 0.124), and the interaction between control attribution and algorithmic procedural fairness was also not significant (*b* = 0.019, *p* = 0.616). After switching to HC3 robust standard errors (Long & Ervin, 2000), the *p*-values for the two interactions were *p* = 0.060 and *p* = 0.698, respectively, neither of which reached the 0.05 significance level. The interaction term for the control attribution path in the first stage was significant under both standard error specifications (*b* = −0.296, *p* < 0.001), whereas the commitment attribution path was sensitive to the standard error specification (*b* = 0.112, *p* = 0.032). To assess the incremental contribution of the second-stage interaction terms, we retained the main effect of algorithmic procedural fairness in the outcome equations of the comparison model and added two interaction terms. The explained variance increased from 0.368 to 0.377. However, this increase did not reach the 0.05 significance level (ΔR^2^ = 0.009, Δ*F*(2, 366) = 2.586, *p* = 0.077; HC3 robust joint test F(2, 366) = 1.856, *p* = 0.158). These results are consistent with the proposed first-stage moderation model; neither the individual second-stage interactions nor their incremental contributions were statistically significant at the 0.05 level.

To test the robustness of the control variable coding, we recoded age, tenure, average daily working hours, education level, and monthly income as ordinal continuous variables and re-estimated the parallel mediation model (Model 4) and the first-stage moderated mediation model (Model 7) using 5000 bootstrap samples. The results of Model 4 indicated that algorithmic gamification was significantly and positively correlated with both commitment attribution (*b* = 0.745, *p* < 0.001) and control attribution (*b* = 0.492, *p* < 0.001). The specific indirect effect through commitment attribution was significantly positive (0.178, 95% CI [0.095, 0.258]), while the specific indirect effect through control attribution was significantly negative (−0.073, 95% CI [−0.115, −0.036]), and the total indirect effect was significantly positive (0.105, 95% CI [0.012, 0.196]). The results of Model 7 showed that the first-stage moderating effect of algorithmic procedural fairness on the control attribution path was significantly negative (*b* = −0.281, *p* = 0.002), with a significantly positive moderated mediation index (0.042, 95% CI [0.016, 0.079]). The first-stage interaction term for the commitment attribution path was significantly positive (*b* = 0.106, *p* = 0.045), and its moderated mediation index was also significant (0.025, 95% CI [0.006, 0.059]). Therefore, the findings of this study demonstrate good robustness.

## 5. Conclusions and Discussion

### 5.1. Conclusions

In the context of gig platforms, this study examines how algorithmic gamification is associated with gig workers’ proactive service behaviour through commitment attribution and control attribution, and how these associations vary with algorithmic procedural fairness. The findings indicate that algorithmic gamification is positively correlated with both commitment and control attribution of gig workers, demonstrating a significant “double-edged sword” effect; furthermore, these two types of attributions coexist rather than offset one another. Second, the indirect effect of the commitment attribution path was numerically greater than that of the control attribution path, resulting in an overall positive indirect effect of algorithmic gamification on gig workers’ proactive service behaviour. Third, algorithmic procedural fairness strengthens the positive effect of the commitment attribution path and weakens the negative effect of the control attribution path. Moreover, when algorithmic procedural fairness is high, the indirect effect of control attribution on proactive service behaviour becomes non-significant.

### 5.2. Theoretical Contributions

First, this study identifies algorithmic gamification as an antecedent of proactive service behaviour and reveals its “double-edged sword” effect, extending research on the antecedents of proactive service behaviour. The existing literature on algorithmic gamification often falls into a binary opposition between “pure empowerment” (improving satisfaction and efficiency) and “algorithmic exploitation” (exacerbating internal friction and dehumanisation) (Cameron, 2024; Z. Zhang et al., 2025). This study extends research on the antecedents of proactive service behaviour from traditional interpersonal management to the context of algorithmic governance (Y. Wu et al., 2025). Furthermore, existing research on algorithmic gamification mostly focuses on quantifiable in-role performance and predominantly reports its positive incentive effects (Cameron, 2022), while research conclusions regarding extra-role proactive service behaviour remain unclear (Z. Zhang et al., 2025). This study reveals the “double-edged sword” impact of algorithmic gamification on proactive service behaviour, echoing the perspective of the commitment–control paradox in gig work (Cameron, 2024).

Second, this study examines commitment attribution and control attribution as distinct but potentially coexisting interpretations of platform intent, extending the focus of gamification research from functional affordances to the meanings workers assign to them. Existing research largely treats control- and commitment-oriented attributions as either substitutive (Arthur, 1994) or independent (Zhou et al., 2025); however, few studies have explored how gamification influences both types of attribution. This study found that algorithmic gamification is positively associated with both commitment and control attribution, consistent with their conceptualisation as distinct, potentially coexisting dimensions rather than opposing poles of a single continuum (Hu et al., 2025; Nishii et al., 2008). Algorithmic gamification possesses a duality of meaning, encompassing both “feedback-recognition” and “evaluation-supervision” whereby gamification amplifies both sets of cues concurrently. Simultaneously, gig workers can identify the platform’s dual objectives of “improving service quality while strengthening labour control,” thereby forming two parallel intentional inferences. This result extends the findings of Van De Voorde and Beijer (2015), who showed that high-performance work systems simultaneously enhance both types of attribution in the context of algorithmic management. Therefore, the two attributions may coexist, while their indirect effects on proactive service behaviour run in opposite directions. The positive indirect effect through commitment attribution was larger in absolute magnitude than the negative indirect effect through control attribution, yielding a positive total indirect effect. This deepens our understanding of the complex behavioural consequences of algorithmic gamification (Cameron, 2024; Santabarbara et al., 2026).

Third, this study examines algorithmic procedural fairness as a boundary condition for the two attribution pathways in platform work settings. Existing research has examined the legitimacy of algorithmic decisions (Martin & Waldman, 2023) and the relationship between organisational fairness and work attitudes (Pinho & Colston, 2025). Although prior studies have suggested that enhancing the fairness of algorithmic decision-making may mitigate the negative impacts of algorithmic management, the moderating role of algorithmic procedural fairness in the context of algorithmic gamification remains to be systematically tested. Procedural fairness signals to gig workers that the platform is willing to constrain its own power and consider the interests of the labourers. This study further demonstrates that algorithmic procedural fairness can strengthen the positive effects of the commitment attribution pathway while weakening the negative effects of the control attribution pathway; notably, the mediating path of control attribution is not significant under conditions of high procedural fairness. This finding responds to the call by Zhou et al. (2025) to examine the moderating role of fairness principles, suggesting that it is difficult for platforms to improve gamification outcomes by “eliminating” control attributions. A more realistic path is to calibrate the relative strength of the two types of attributions through diagnostic cues.

### 5.3. Managerial Implications

First, platforms should convey credible supportive intentions through gamified design to strengthen gig workers’ commitment attribution. This study found that the positive effects of algorithmic gamification are primarily realised through commitment attribution, and the indirect effect of this path is larger in absolute magnitude than that of the control attribution path. At the interface level, platforms can disclose the scope and purpose of data recording to gig workers and provide brief rule explanations for dispatching and rating results, making the platform’s supportive intentions identifiable rather than merely speculative. Platforms can also recognise less readily quantified forms of proactive service through customer feedback tags, examples of good service, and periodic feedback on service contributions. At the rules level, evaluation criteria can extend beyond order volume and completion speed to include customer satisfaction, problem prevention, and long-term service contributions. Human review can help ensure that additional efforts are recognised in formal evaluations and rewards. Supportive design should extend beyond interface wording: when promised rewards are not delivered consistently, visible recognition may instead reinforce the interpretation that the platform is seeking additional effort.

Second, platforms should treat weakening control attribution as an independent management objective, rather than expecting it to disappear automatically as commitment attribution increases. This study shows that algorithmic gamification is not merely a technical incentive tool; it can also trigger control attribution among gig workers. Accordingly, at the interface level, leaderboards could be set by default to “visible only to the individual” or limited to small groups within the same city and at the same level, with an option to disable them if desired; showing gig workers their progress over time rather than their rankings relative to others may reduce competitive interpretations. Challenge tasks should be presented with “Accept/Skip” options side by side; skipping should leave no record, trigger no subsequent prompts, and be clearly labelled in the interface as having no impact on basic task allocation. Gamification rewards should provide additional benefits and not be tied to punitive consequences, such as demotion or restrictions on task assignments. At the rules level, gig workers’ intention attribution should be incorporated into the functional evaluation system, with brief surveys conducted before and after the launch of gamification features to monitor levels of control attribution, thereby making this cost visible, which is otherwise hidden in output metrics.

Third, platforms should establish clear procedural safeguards, including meaningful transparency and mechanisms for correcting their decisions. Algorithmic procedural fairness may strengthen the association between algorithmic gamification and commitment attribution while weakening its association with control attribution. First, information disclosure should be centred on fairness; platforms should explain to gig workers the criteria used for dispatching, advancement, rewards, and performance evaluations, how consistency is guaranteed for everyone, and how deviations are corrected, rather than simply providing technical details. Second, platforms should establish accessible and responsive appeal mechanisms, provide human reviews when necessary, and use appeal outcomes to revise rules. These measures can make the possibility of correcting decisions tangible for gig workers. Finally, regular algorithmic fairness audits should be conducted to compare whether systematic differences exist among new and veteran workers, full-time and part-time workers, and groups in different service areas regarding task opportunities, ranking results, and reward acquisition. Gamification rules should be continuously revised based on audits and grievance results. It should be noted that building institutions for procedural fairness takes time, whereas gamification mechanisms are often launched first. For platforms where procedural fairness is not yet mature, the intensity of gamification should be adjusted downward accordingly: while an appeal mechanism is being developed, platforms can provide concise explanations of dispatch and rating decisions as an interim transparency measure. Additionally, horizontal comparisons on leaderboards should be temporarily disabled, the frequency of order-rushing prompts reduced, and dynamic thresholds suspended, to be gradually restored only after the level of procedural fairness has improved.

### 5.4. Limitations and Future Research Directions

First, although this study used a three-wave time-lagged design to separate measurements over time and help reduce common method bias, all variables were self-reported by the participants. Future research could incorporate data from multiple sources and use scenario-based experiments or quasi-experimental designs to strengthen causal inference. Second, the sample in this study primarily consisted of gig workers from food delivery, ride-hailing, and crowdsourced instant-delivery platforms; whether the conclusions can be generalised to other types of platforms, such as crowdsourced design or freelance outsourcing, remains to be tested. Third, this study analysed the mechanism by which algorithmic gamification affects gig workers’ proactive service behaviour based on attribution theory. Future research could further explore mediating mechanisms from other theoretical perspectives, such as resource conservation theory. Fourth, this study measured algorithmic gamification as an overall perception and did not distinguish the effects of specific game elements, such as challenge, feedback, competition, social interaction, and rewards. Future research could utilise multidimensional scales or configurational approaches for a more granular analysis. Fifth, this study examined algorithmic procedural fairness as a moderator of the first stage of the mediation process. Future research could examine whether factors such as time pressure moderate the second stage.

## Figures and Tables

**Table 1:** Demographic profile of the sample.

Variable	Category	Number	Percentage	Variable	Category	Number	Percentage
Gender	Male	305	77.8%	Education	Below junior college	40	10.2%
	Female	87	22.2%		Junior college	126	32.1%
Age	Under 20	16	4.1%		Bachelor’s degree	208	53.1%
	20–30	166	42.3%		Postgraduate	18	4.6%
	31–40	172	43.9%	Daily working hours	Within 4 h	67	17.1%
	Over 40	38	9.7%		4–8 h	120	30.6%
Platform type	Food-delivery riders	133	33.9%		8–12 h	181	46.2%
	Ride-hailing drivers	131	33.4%		Over 12 h	24	6.1%
	Crowdsourced instant-delivery couriers	128	32.7%	Monthly income (RMB)	1000–3000	62	15.8%
Tenure	1–3 months	40	10.2%		3000–5000	83	21.2%
	3–6 months	56	14.3%		5000–8000	135	34.4%
	6 months–1 year	104	26.5%		Above 8000	112	28.6%
	1–2 years	120	30.6%				
	Over 2 years	72	18.4%				

**Table 2:** Confirmatory factor analyses.

Model	χ^2^	*df*	χ^2^/*df*	CFI	TLI	SRMR	RMSEA
Five-factor model + CMV factor	430.822	338	1.275	0.989	0.986	0.025	0.027
Five-factor model	503.712	367	1.373	0.983	0.982	0.033	0.031
Four-factor model (a)	1028.162	371	2.771	0.920	0.913	0.085	0.067
Four-factor model (b)	1537.278	371	4.144	0.858	0.845	0.082	0.090
Three-factor model	2116.184	374	5.658	0.788	0.770	0.126	0.109
One-factor model	4044.593	377	10.728	0.554	0.520	0.146	0.158

Note: *N* = 392. The four-factor model (a) combines commitment attribution and proactive service behaviour, while the four-factor model (b) combines commitment attribution and control attribution into a single factor. The three-factor model combines commitment attribution, control attribution, and proactive service behaviour. CMV is an unmeasured common-method variance factor.

**Table 3:** Descriptive statistics and correlations.

	1	2	3	4	5
1 Algorithmic gamification	(0.826)				
2 Commitment attribution	0.622 **	(0.831)			
3 Control attribution	0.320 **	0.184 **	(0.914)		
4 Proactive service behaviour	0.474 **	0.461 **	−0.035	(0.775)	
5 Algorithmic procedural fairness	0.457 **	0.461 **	0.080	0.432 **	(0.774)
Mean	4.085	3.824	3.783	3.961	4.065
SD	0.682	0.837	1.097	0.722	0.676

Note: ** *p* < 0.01; values in parentheses on the diagonal are the square roots of the AVEs.

**Table 4:** Results of the regression and moderation analyses.

	Commitment Attribution				Control Attribution				Proactive Service Behaviour		
	M1	M2	M3	M4	M5	M6	M7	M8	M9	M10	M11
**Control variables**											
Gender	Yes	Yes	Yes	Yes	Yes	Yes	Yes	Yes	Yes	Yes	Yes
Platform type	Yes	Yes	Yes	Yes	Yes	Yes	Yes	Yes	Yes	Yes	Yes
Age	Yes	Yes	Yes	Yes	Yes	Yes	Yes	Yes	Yes	Yes	Yes
Education	Yes	Yes	Yes	Yes	Yes	Yes	Yes	Yes	Yes	Yes	Yes
Tenure	Yes	Yes	Yes	Yes	Yes	Yes	Yes	Yes	Yes	Yes	Yes
Daily working hours	Yes	Yes	Yes	Yes	Yes	Yes	Yes	Yes	Yes	Yes	Yes
Monthly income	Yes	Yes	Yes	Yes	Yes	Yes	Yes	Yes	Yes	Yes	Yes
Algorithmic gamification		0.734 ***	0.610 ***	0.650 ***		0.436 ***	0.461 ***	0.354 ***		0.500 ***	0.393 ***
Commitment attribution											0.232 ***
Control attribution											−0.145 ***
Algorithmic procedural fairness			0.267 ***	0.280 ***			−0.054	−0.088			
Algorithmic gamification × Algorithmic procedural fairness				0.112 *				−0.296 ***			
Constant	3.441 ***	3.721 ***	3.772 ***	3.814 ***	2.893 ***	3.059 ***	3.049 ***	2.939 ***	3.499 ***	3.690 ***	3.270 ***
*R* ^2^	0.133	0.459	0.492	0.499	0.164	0.231	0.232	0.258	0.063	0.267	0.342
Adjusted *R*^2^	0.089	0.430	0.464	0.469	0.122	0.190	0.189	0.213	0.015	0.227	0.303
*F*	3.000 ***	15.757 ***	17.097 ***	16.693 ***	3.847 ***	5.582 ***	5.326 ***	5.823 ***	1.310	6.746 ***	8.712 ***
Δ*F*		223.968 ***	24.199 ***	4.655 *		32.372 ***	0.385	12.724 ***		103.191 ***	21.073 ***
*N*	392	392	392	392	392	392	392	392	392	392	392

Note: *N* = 392; * *p* < 0.05, *** *p* < 0.001.

**Table 5:** Parallel mediation analysis.

Effect	Estimate	Boot *SE*	95% CI
Total indirect effect (|commitment attribution path| − |control attribution path|)	0.107	0.046	[0.008, 0.193]
Algorithmic gamification → commitment attribution → proactive service behaviour	0.170	0.042	[0.083, 0.248]
Algorithmic gamification → control attribution → proactive service behaviour	−0.063	0.019	[−0.103, −0.028]

Note: *N* = 392; 5000 bootstrap resamples; percentile confidence intervals; all equations include 19 control dummy variables. Because the two specific indirect effects have opposite signs, the total indirect effect equals the difference between their absolute values; its confidence interval tests whether the positive effect via commitment attribution is greater in absolute value than the negative effect via control attribution.

**Table 6:** Moderated mediation analysis.

Mediator	Algorithmic Procedural Fairness	Estimate	Boot *SE*	95% [LLCI, ULCI]
Commitment attribution	M − SD	0.133	0.034	[0.064, 0.198]
	M	0.151	0.037	[0.074, 0.221]
	M + SD	0.169	0.043	[0.082, 0.250]
	Index of moderated mediation	0.026	0.016	[0.001, 0.063]
Control attribution	M − SD	−0.080	0.023	[−0.129, −0.039]
	M	−0.051	0.019	[−0.093, −0.017]
	M + SD	−0.022	0.021	[−0.067, 0.017]
	Index of moderated mediation	0.043	0.015	[0.019, 0.080]

Note: A confidence interval that does not contain zero indicates a significant effect.

**Table 7:** Measurement invariance test.

Model	χ^2^	df	χ^2^/df	CFI	TLI	RMSEA	SRMR
M1 Configural invariance	1455.774	1101	1.322	0.958	0.953	0.050	0.050
M2 Metric invariance	1513.373	1149	1.317	0.957	0.954	0.049	0.059
M3 Scalar invariance	1573.965	1197	1.315	0.955	0.954	0.049	0.060
Model comparison	Δχ^2^	Δdf	*p*	ΔCFI	ΔTLI	ΔRMSEA	ΔSRMR
M2 vs. M1	57.600	48	0.162	−0.0011	0.0007	−0.0004	0.0085
M3 vs. M2	60.592	48	0.105	−0.0015	0.0003	−0.0002	0.0013

**Table 8:** Multi-group analysis results.

Path	Food-Delivery Riders	Ride-Hailing Drivers	Crowdsourced Instant-Delivery Couriers	Tests of Path Equality F(2, 363)	*p* (HC3)
Algorithmic gamification → Commitment attribution	0.732 ***	0.438 **	0.857 ***	2.714	0.068
Algorithmic procedural fairness → Commitment attribution	0.313 *	0.315 **	0.190	0.278	0.757
Algorithmic gamification × Algorithmic procedural fairness → Commitment attribution	0.171	0.035	0.089	0.118	0.889
Algorithmic gamification → Control attribution	0.211	0.456 *	0.311	0.468	0.627
Algorithmic procedural fairness → Control attribution	0.010	0.053	−0.368 *	1.733	0.178
Algorithmic gamification × Algorithmic procedural fairness → Control attribution	−0.165	−0.283	−0.596 **	0.889	0.412
Algorithmic gamification → Proactive service behaviour	0.558 ***	0.242	0.401 *	1.114	0.329
Commitment attribution → Proactive service behaviour	0.235 *	0.423 ***	0.121	1.772	0.172
Control attribution → Proactive service behaviour	−0.087	−0.144 ***	−0.171 ***	0.608	0.545

Note: HC3 = heteroskedasticity-consistent standard error estimator; * *p* < 0.05, ** *p* < 0.01, *** *p* < 0.001.

## Data Availability

The data presented in this study are openly available in OSF at https://osf.io/k4u7c/files/osfstorage.

## Appendix A

**Table A1:** Measurement scales, reliability and validity.

Construct	Measurement Items	Factor Loading
Algorithmic gamification	Algorithmic gamification (α = 0.950; CR = 0.951; AVE = 0.682)	
	The platform pushes challenging tasks to me based on my order records and ratings	0.815
	The platform automatically updates my ranking or level based on my algorithm-recorded service performance	0.811
	The platform marks my work achievements with badges, levels, milestones and the like	0.842
	The platform has me compete with other workers on performance through leaderboards or team contests	0.840
	The platform provides interactive mechanisms for gig workers to form teams, help one another and share progress	0.823
	The platform encourages me to exchange experience with other gig workers in its online community	0.830
	The platform presents dispatched tasks to me in the form of “quests” or “challenges”	0.832
	On the platform I can view my task progress and goal attainment in real time	0.826
	When I am close to an order-volume target or a tiered reward, the platform prompts me with “how far I still have to go”	0.811
Commitment attribution	Commitment attribution (α = 0.869; CR = 0.870; AVE = 0.690)	
	The platform adopts gamification to help me perform my work better and provide customers with high-quality service	0.822
	The platform adopts gamification to help me improve my work capabilities and better accomplish platform tasks	0.824
	The platform adopts gamification to improve my work experience and make me feel that my labour is valued and recognised	0.846
Control attribution	Control attribution (α = 0.937; CR = 0.938; AVE = 0.835)	
	The platform adopts gamification to reduce labour and management costs and improve operational efficiency	0.919
	The platform adopts gamification to monitor more closely whether I comply with platform rules and requirements	0.904
	The platform adopts gamification to induce me to invest more labour and thereby obtain greater labour output for the platform	0.919
Proactive service behaviour	Proactive service behaviour (α = 0.913; CR = 0.913; AVE = 0.601)	
	I proactively share information with customers to meet their needs	0.791
	I anticipate problems or needs that customers may have and proactively propose solutions	0.818
	I proactively adjust my service approach according to the specific service situation and potential risks	0.692
	I proactively take responsibility for the service and keep in close communication with customers to ensure its smooth completion	0.789
	I proactively coordinate and cooperate with other service staff so as to better meet customer needs	0.754
	I proactively communicate with customers about their service needs and help resolve related problems	0.749
	I proactively seek customer feedback to confirm whether their needs and expectations have been met	0.826
Algorithmic procedural fairness	Algorithmic procedural fairness (α = 0.911; CR = 0.912; AVE = 0.599)	
	The platform algorithm follows consistent rules when dispatching orders	0.783
	The platform algorithm evaluates my service performance accurately	0.814
	The platform algorithm shows no obvious bias in rankings, rewards and sanctions	0.785
	The platform algorithm applies consistent and fair evaluation and treatment standards to different gig workers	0.841
	The platform algorithm explains the main rules affecting order dispatching, rankings or rewards	0.703
	When I consider an algorithmic decision unreasonable, the platform allows me to appeal	0.759
	Based on appeals or feedback, the platform reviews and corrects unreasonable algorithmic decisions	0.725
